# Use of KIDSCREEN health-related quality of life instruments in the general population of children and adolescents: a scoping review

**DOI:** 10.1186/s12955-023-02088-z

**Published:** 2023-01-20

**Authors:** Eva-Grethe Befus, Sølvi Helseth, Eirin Mølland, Thomas Westergren, Liv Fegran, Kristin Haraldstad

**Affiliations:** 1 Faculty of Health- and Sport Sciences, University of Agder, P.O. Box 422, 4604 Kristiansand, Norway; 2 Faculty of Health, OsloMet – Oslo Metropolitan University, St. Olavs Plass, P.O. Box 4, 0130 Oslo, Norway; 3 Department of Economics and Finance, School of Business and Law, University of Agder, P.O. Box 422, 4604 Kristiansand, Norway; 4 Faculty of Health Sciences, University of Stavanger, P.O. Box 8600, 4036 Stavanger, Norway

**Keywords:** Kidscreen, Quality of life, Health-related quality of life, Scoping review

## Abstract

**Purpose:**

Subjectively assessing health related quality of life (HRQoL) in children and adolescents is increasingly important in the public health field. One valid and widely used generic HRQoL instrument is the KIDSCREEN questionnaire. The aim of this study was to map all studies using KIDSCREEN instruments in the general population of children and adolescents aged 6–18 years.

**Methods:**

A scoping review was conducted. The search strategy was formulated according to the Preferred Reporting Items for Systematic Reviews and Scoping Reviews guidelines. The databases Cinahl, socINDEX, Medline, Embase, APA Psychinfo, Scopus, and Eric were searched in October 2021.

**Results:**

In total, 1365 papers were eligible for screening, 1031 were excluded and 334 reports were read in full. 252 reports were included. KIDSCREEN studies in the general population was predominantly conducted in Europe (n = 211). Most studies (n = 179) had a cross sectional design, while few experimental studies (n = 24) were found. The three KIDSCREEN versions comprising of 10, 27 and 52 items, were equally distributed between studies. The self-reported version (n = 225) of the KIDSCREEN instrument was more prevalent than the proxy version, while few studies discussed a cut point. Study contexts reflected international trends of public health challenges, commonly including mental- and psychosocial health, physical activity, socioeconomic status, and obesity.

**Conclusion:**

KIDSCREEN is widely used in cross sectional studies assessing common public health challenges. Experimental and longitudinal assessments, possibly including relevant cut offs remain mainly unexplored and are recommended for future research.

**Supplementary Information:**

The online version contains supplementary material available at 10.1186/s12955-023-02088-z.

## Introduction

Good health-related quality of life (HRQoL) in children and adolescents is crucial for a healthy transition to adulthood [1]. The age range from 10 to 18 years is characterized by major changes, vast growth, and psychological development linked to the extensive individual, cognitive, social, and contextual changes that develop [2, 3]. A systematic assessment of HRQoL in the general population of children and adolescents is important to be able to identify children and subgroups that might be at risk of poor HRQoL [4, 5]. Assessments of HRQoL can support the development and evaluation of public health interventions and can be used for population overview and research [6, 7]. Assessing HRQoL among children and adolescents is crucial in the trajectory of fulfilling the United Nations Sustainable Development Goal 3 of ensuring good health and improving HRQoL for all [7, 8]. Few population-based studies on children and adolescents’ HRQoL have been carried out [5, 9], particularly in the 6–12 year age range [5]. However, numerous studies on cancer and chronic illnesses have been conducted [5], often involving disease-specific HRQoL instruments assessing domains particularly challenged by illness [7]. Furthermore, in the last few years, HRQoL has become a major health outcome in the public health area [10, 11].

HRQoL is described as a subjective term and multidimensional construct including physiological, psychological, and functional aspects of general well-being [12]. The subjective measure provides information on what children and adolescents are experiencing and how they are managing their life [5, 7]. This represents an evident shift in the last decade—the transition from using objective measures to asking children about their subjective well-being, indicating what is important to them [5, 7]. This increased recognition has led to a growing use of children’s self-report HRQoL instruments [5], and it has been established that children older than 8 years can adequately report on their subjective health [5, 13]. Hence, proxy versions of an instrument that involves parents answering on behalf of the children may be used as a supplemental source of information [13] when children are too young or disabled to adequately self-report [14].

To determine HRQoL in the general population of children and adolescents validated and reliable instruments are required [5]. The World Health Organization (WHO) states that instruments should be child centered, age appropriate and should depend on subjective self-report or proxy-reported measures [14]. One valid and widely used generic HRQoL instrument is the KIDSCREEN questionnaire [11], which was chosen for this review. KIDSCREEN was the first HRQoL instrument for children and adolescents to be developed simultaneously in several European countries and further tested in a large sample of children and adolescents [15]. The instrument has shown adequate psychometrics [11], and there are three versions, all available as a self-report and proxy option for parents [13, 16]. The long version, KIDSCREEN 52, is recommended for research purposes and when detailed information on HRQoL is needed. It consists of the following 10 HRQoL dimensions: *Physical Well-being, Psychological Well-being, Moods and Emotions, Self-Perception, Autonomy, Parent Relation and Home Life, Financial Resources, Social Support and Peers, School Environment*, and *Social Acceptance (Bullying)* [17]. KIDSCREEN 27 is a shorter version, and it represents the 10 authentic dimensions condensed into the following 5 dimensions: *Physical Well-being, Psychological Well-being, Autonomy and Parent Relation, Social Support & Peers, and School Environment* [17]. KIDSCREEN 10 provides a global HRQoL score and is recommended for use in large studies [17]. All KIDSCREEN questionnaires may be used appropriately for healthy and ill children and adolescents from 8 to 18 years of age [16]. Proxy versions of the instrument are used from 6 years of age [18]. Previous studies have shown that the instrument is both reliable and valid in measuring HRQoL in children and adolescents [15, 19].

For the purpose of interpreting HRQoL scores on an individual level, specific training for healthcare professionals is necessary [7]. The KIDSCREEN manual provides an interpretation of the KIDSCREEN score, and Hirschfeld and Thiele [20] published a study aimed at finding an optimal cut point for KIDSCREEN 10 and suggested recommended cut points for the questionnaire in the 7–17-year age range. However, no agreement has been established regarding cut points for any of the KIDSCREEN questionnaires [21]. Cut points may assist in the interpretation of individual HRQoL outcomes [20].

To the best of our knowledge, the application of KIDSCREEN instruments in studies of children and adolescents in the general population have not previously been systematically reviewed. However, the KIDSCREEN instrument is increasingly being used in public health- and large-scaled population-based studies. Hence, the need for a review of how the instrument has been used in this context is highlighted. The aim of the present scoping review was to provide an overview of and map studies using KIDSCREEN in the general population of children aged 6–18 years, as well as to describe the country of origin, the study design, whether HRQoL was a main focus, the version of KIDSCREEN instrument(s) being used, the age group, if a cut point for KIDSCREEN was discussed, and the study context.

## Methods

This review was designed as a scoping review, as defined by Peters et al. [22]. We used the Preferred Reporting Items for Systematic Reviews and Meta-Analyses extension for scoping reviews checklist (PRISMA-ScR) [23] to provide precision and facilitate transparent and complete reporting of this scoping review.

### Eligibility criteria

The review process followed a preplanned unpublished protocol. The inclusion and exclusion criteria were developed a priori.

### Inclusion criteria

Quantitative primary reports were included if they were performed using KIDSCREEN instruments on a sample from the general population, peer reviewed, and published in the English language. The general population was defined as all individuals aged 6–18 years old recruited from a population with no specific disease or clinical condition, regardless of the study outcome.

### Exclusion criteria

Reports were excluded if they included a population derived from a clinical setting (understood as a hospital, department, or outpatient facility) or if the study sample was characterized by a diagnosis or condition. Reports that included a healthy control group were excluded. Conference abstracts, validation or methodological reports, editorials, opinion articles, scientific statements, guidelines, protocols, and review studies were excluded (Additional file 1).

### Search strategy

Systematic literature searches for publications using KIDSCREEN instruments were conducted in collaboration with a trained librarian. On October 21st, 2020, the searches were performed on the following databases: CINAHL (EBSCOhost), SocINDEX (EBSCOhost), MEDLINE (Ovid), Embase (Ovid), APA PsycINFO (Ovid), and Scopus. To ensure coverage of all KIDSCREEN reports, the search term used was KIDSCREEN* OR (kid OR kids) adj2 screen* (Ovid). We searched for peer-reviewed publications published after January 1st, 2000, and in the English language, as the KIDSCREEN instruments did not exist prior to this time. On October 13th, 2021, the same search strategy was performed in the Eric database to include education-related reports. On October 22nd, 2021, an updated search, identical to the first search, was conducted to include the most recent reports. Gray literature was not queried. A full overview of the search terms can be found in the Additional file 2.

### Study selection process

Six trained researchers participated in the screening process, and using the Rayyan online screening tool, two reviewers screened all papers independently by title and abstract [24]. The first author screened all papers to ensure consistency. If “KIDSCREEN” was not mentioned in the title or abstract but “quality of life,” QoL, HRQoL, or “well-being” was present, the full text was searched for KIDSCREEN instruments to ensure the inclusion of all relevant reports. Reports eligible for full-text screening were read in full, independently, by two reviewers who worked in pairs. The first author read all reports. In the case of disagreements or uncertainties, consensus was achieved by all six reviewers through discussion.

### Data collection process and data items

A data-charting form to register the key characteristics of the reports was jointly developed by all reviewers. In accordance with the aim of the study, we reviewed the included publications in terms of country, study design, if HRQoL was a main focus, KIDSCREEN instrument(s) used, if self or proxy measurements were used, number of participants, age groups, study context, and whether a cut point for KIDSCREEN was discussed. Data was extracted, independently, in pairs; the first author extracted data from all reports. Variables measured in relation to HRQoL were categorized in groups by two reviewers.

## Results

### Study selection

**Fig. 1:**
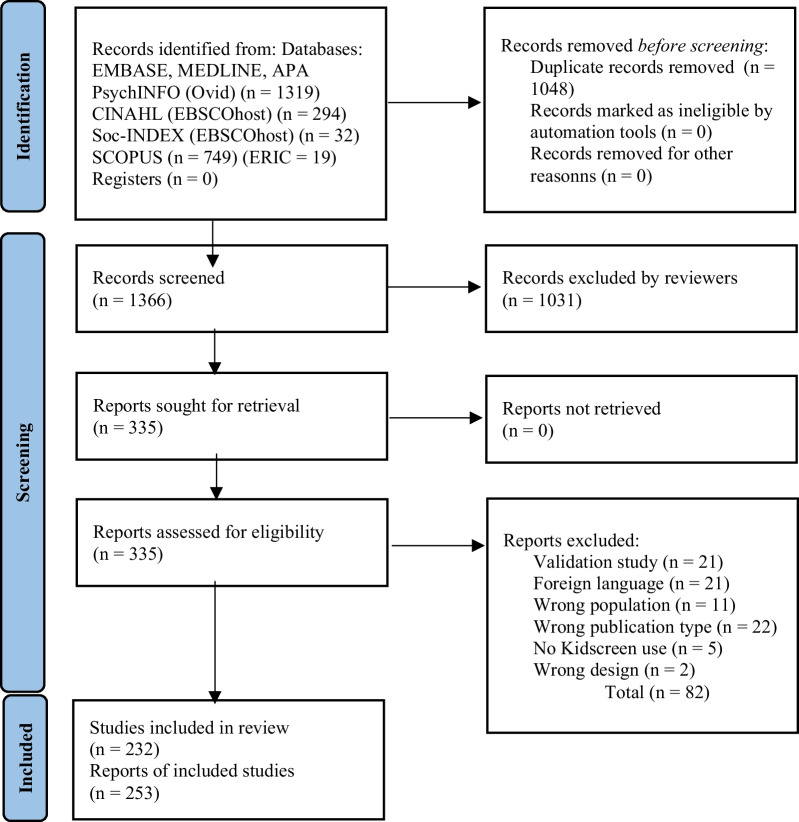
Flow chart of inclusion. *Source*: Page MJ, McKenzie JE, Boutron I, Hoffmann TC, Mulrow CD, et. al. The PRISMA 2020 statement: an updated guideline for reporting systematic reviews. BMJ 2021;372:n71. https://doi.org/10.1136/bmj.n71

The literature searches resulted in 2414 publications. After removing duplicates, 1366 papers were eligible for screening. In total, 1031 publications were excluded during the screening process. The remaining 335 publications were read in full, independently, and 253 reports from 232 studies were finally included in the review, described according to the 2020 Prisma flow chart of inclusion. Reasons for the 82 full texts excluded, and a flowchart detailing the study selection and inclusion is given in Fig. 1.

### Study characteristics

The studies included in this review were conducted all over the world—with the most articles published in Spain, followed by Portugal. Several European countries follow, and if taken as a whole, more articles have been produced in Europe (n = 211) than in the rest of the world. In South America, Asia, Africa, North America, Oceania, and Central America, combined, 60 publications have been produced, and as few as 6 studies have been published in North America (Table 1).

**Table 1:** Number of occasions Kidscreen has been applied in different countries across the included studies (n = 252)

Country where Kidscreen was studied	N
*Europe*	*211*^a^
Spain	49
Portugal	34
Germany	26
Netherlands, Norway	17
Sweden	16
England, Poland, Switzerland, United Kingdom	15
Greece	14
Austria	11
France	10
Ireland	7
Hungary, Italy	6
Czech Republic, Northern Ireland	5
Finland, Denmark	3
Belgium, Scotland	2
Bulgaria, Croatia, Latvia, Serbia, Slovenia, Turkey	1
*South America*	*21*
Brazil	10
Columbia	6
Argentina	5
Chile	3
Peru	1
*Asia*	*18*
India	6
Iran	4
China	4
Hong Kong	2
Indonesia, Jordan, Lebanon, South Korea, Philippines	1
*Africa*	*6*
South Africa	6
Kenya	3
*North America*	*6*
Canada	4
USA	4
Mexico	1
*Oceania*	*8*
Australia	8
*Central America*	*1*
Panama	1

The table presents number of occasions Kidscreen has been applied in each country across the 252 included studies. Nine studies were conducted in Europe across several countries (Austria, Czech Republic, France, Germany, Greece, Hungary, Poland, Spain, Switzerland, Sweden, the Netherlands, and United Kingdom) 1 study was conducted across Spanish-speaking countries (Argentina, Chile, Colombia, Mexico, Panama, Spain), and 3 studies were conducted across countries on all continents, except Antarctica (Australia, Canada, China, Colombia, England, Finland, India, Kenya, Portugal, and United States of America). All, except the European studies, were conducted in both developed and developing countries

^a^One single study may have been conducted in several different countries. We include all countries where the study has been conducted; therefore, the sum exceeds total number of included studies (n = 253)

The predominant design was cross-sectional/descriptive and was used in 71.0% of the included reports, 16.3% reports had a cohort/prospective/longitudinal design, 9.5% were randomized controlled trials (RCTs), 2.8% had a quasi-experimental design, and 0.4% were case–control studies (Table 2). The majority (77%) of the studies recruited participants solely from a school setting, while 23% of the studies recruited from other settings, such as sampling from a municipal population registration, telephone sampling, or birth cohort sampling. HRQoL as a main focus was defined by HRQoL, QoL, well-being, or wellness being present in the title of cross-sectional, longitudinal, quasi-experimental, and case–control studies. For RCTs, HRQoL as a main outcome was described. HRQoL was the main outcome in 70.8% of the RCTs, and for the remaining study designs, 78.0% of the reports used HRQoL as a main focus.

**Table 2:** Study design and HRQoL as main outcome

Design	N (%)	HRQoL as main outcome (% among study category)	HRQoL as main focus (% among study category)
Cross-sectional/descriptive	180 (71.0)		144 (80.0)
Cohort/prospective/longitudinal	41 (16.3)		31 (75.6)
RCT/experimental	24 (9.5)	17 (70.8)	
Quasi-experimental	7 (2.8)		3 (42.8)
Case–control	1 (0.4)		1 (100)
Total	253	17	179 (78)

### KIDSCREEN version used

Table 3 shows a nearly equal distribution between the use of the three KIDSCREEN instruments. Twelve percent of the reports calculated a global HRQoL score, using KIDSCREEN 10 in addition to KIDSCREEN 27 or KIDSCREEN 52, but were characterized according to the longest version (Table 3). Self-reported measurements were used alone in 89% of the reports reviewed, while 4% of the reports solely used proxy versions. Seven percent of the reports combined self-report and proxy versions of the KIDSCREEN instrument. As few as 5% of the publications used a cut point for one or several dimensions of the KIDSCREEN instrument. Of these 13 publications, one defined a cut point for KIDSCREEN 10 [25]. This cut point was developed for use in epidemiological studies and clinical trials, not for individual diagnosis. Cut points in the remaining 12 reports [26–37] were discussed according to the KIDSCREEN manual [17].

**Table 3:** Characteristics of included studies according to KIDSCREEN version, cut point, age range and gender

Study characteristics	N (%)	Only self-reported n (%)	Only proxy-reported n (%)	Self and proxy n (%)
Total number of included studies	253	226 (89)	10 (4)	17 (7)
Kidscreen 10	92 (36)	82 (89)	4 (4)	6 (7)
Kidscreen 27	80 (31)	70 (88)	5 (6)	5 (6)
Kidscreen 52	82 (33)	74 (90)	1 (1)	7 (9)
Cutpoint used for Kidscreen	13 (5)	11	1	1
6–7 age range	20 (8)	12 (60)	3 (15)	5 (25)
8–11 age range	150 (59)	127 (85)	7 (5)	16 (11)
12–15 age range	186 (74)	166 (89)	5 (3)	15 (8)
16–18 age range	127 (50)	110 (87)	2 (2)	15 (12)
Total number of girls in all studies combined	352,410 (52)	–	–	
Total number of boys in all studies combined	325,992 (48)	–	–	

Age range: The age range data was extracted as it was written in each study, i.e., 6–12, 11–16, or 12–14. One study may be placed in one to four age ranges. As a result, the sum of these will exceed the total number of studies. 5 studies included participants under 6 years, while 19 studies included participants over 18 years. 33 studies did not report the age group of the participants

### Age range of participants

The 8–11 and 12–15 age ranges were included in the majority (59% and 74%, respectively) of the studies, while 50% of the studies included adolescents aged 16–18. As few as 8% of the studies included the 6–7 age range. The age range data were extracted as it was written in each study (i.e., 6–12, 11–16, or 12–14). Hence, the same study might have been placed in one to four age-range categories. As a result, the sum of these will exceed the total number of reports (n = 253). Five reports included participants under 6 years, while 19 reports included participants over 18 years. A total of 178 reports included more than one age range. Overall, the study participants included 52% girls, and the number of study participants ranged from 31 in the study with the lowest number of participants to 164,580 in the study with the highest number of participants (Table 4). Each KIDSCREEN instrument (10, 27, and 52) was used both in small studies and in large population-based studies.

**Table 4:** Study population within studies regarding KIDSCREEN instruments

KIDSCREEN instrument	Sample range	Mean	Median
KIDSCREEN 10	31–164,580	3308	842
KIDSCREEN 27	85–12,494	3250	844
KIDSCREEN 52	32–76,229	3215	840

### Variables measured related to KIDSCREEN

**Fig. 2:**
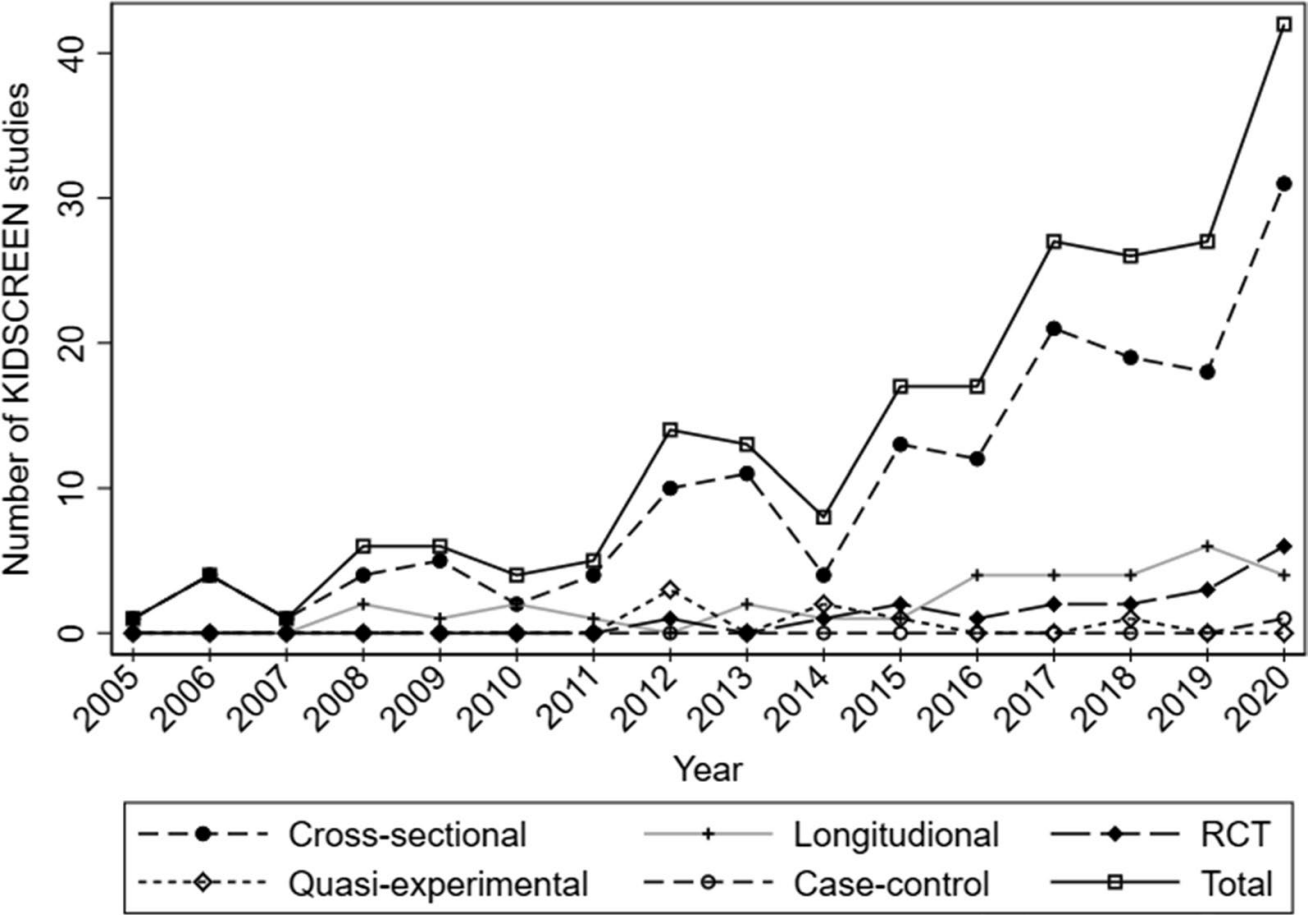
Number of KIDSCREEN studies and study designs conducted in the general population between 2005 and 2020 (n = 253). Source: STATA (StataCorp. 2019, Stata Statistical Software: Release 16. College Station, TX, USA)

An overview of the different conditions and topics measured in relation to KIDSCREEN are found in Table 5. Within the included reports, the most prevalent conditions/topics assessed in relation to HRQoL were mental health/psychosocial health (41%), physical activity (27%), socioeconomic status (SES) (24%), obesity/body mass index (BMI) (19%), school/academic performance (13%), family relations (11%), and screen time/gaming/internet use (10%) (Table 5). In recent years, there has been an increase in KIDSCREEN studies. In 2005, only one report was published, while 46 KIDSCREEN reports were published in 2020. Figure 2 shows this trend for all KIDSCREEN reports, including the various study designs of reports published between 2005 and 2020. Cross-sectional reports have seen the greatest increase, while 63% of the RCT studies were conducted from 2019 onward. Longitudinal reports have also increased, with 49% published from 2019 onward.

**Table 5:** Conditions/topics^a^ assessed in relation to KIDSCREEN in the included studies (n = 252)

Conditions/topics	N (%)
Mental health/psychosocial health	104 (41%)
Physical activity	69 (27%)
Socioeconomic status	61 (24%)
Obesity/BMI (body mass index)	48 (19%)
School/academic performance	32 (13%)
Family relations	28 (11%)
Screen time/gaming/internet-use	26 (10%)
Social support	22 (9%)
Maturity, Nutrition	19 (8%)
Bullying	18 (7%)
Chronic health conditions^b^	15 (6%)
Substance use, Sleep, Immigrants	12 (5%)

^a^The table presents number of occasions each condition/topic has been assessed in relation to KIDSCREEN. One single study may have included various conditions/topics. We included all conditions/topics assessed in relation to KIDSCREEN. The total will, therefore, exceed total number of included studies and is therefore not calculated. The conditions/topics occurring less than 12 times, are not mentioned in the table. Examples of topics not mentioned: Religious practice, connection to pets, adverse childhood experience, connection to nature, cut point development, Covid 19, and youth in foster care

^b^Chronic health conditions, not including obesity and mental illnesses. Examples of chronic health conditions: Asthma, celiac disease, headache, and back pain. The presence of a chronic health condition was measured in participants recruited from the general population

## Discussion

This review was conducted to identify all reports using KIDSCREEN instruments in the general population of children and adolescents aged 6–18 years. The findings revealed that KIDSCREEN studies in the general population were performed all over the world, used different research designs, mainly assessed HRQoL as a main outcome or focus, and involved a variety of conditions and topics related to HRQoL. Furthermore, most papers applied self-reporting of HRQoL, and few of the included reports discussed a cut point for the KIDSCREEN instrument.

Most of the KIDSCREEN studies were conducted in Europe—which is not surprising, as the instrument was developed in Europe through the European Commission–funded KIDSCREEN project conducted in several European countries [17]. Thirteen countries were involved, and the instrument was established in each country. Subsequently, it has been translated, validated, and used successfully in countries outside Europe [38–51]. Our findings likewise indicate that the KIDSCREEN instrument is used cross-nationally, as 9 studies were conducted across several European countries, 3 studies were carried out across countries on all continents, except Antarctica, and 1 study was carried out across Spanish-speaking countries. All, except the European studies, included both developed and developing countries. This may suggest that the instrument works well across countries. Solans et al. [52] argued that the development of the KIDSCREEN instrument across several countries promotes its use and comparability in international studies and that it maintains content validity across different languages. When measuring subjective HRQoL, it is essential to comprehend the cultural context in which a child lives [5, 7], as it affects how HRQoL questions are interpreted, and how the HRQoL concept itself is understood [7]. We also found population-based KIDSCREEN studies performed outside Europe, supporting international usability of the instrument. Internationally, KIDSCREEN is currently a recommended tool for HRQoL assessment by the International Consortium for Health Outcomes Measurement [53].

Even with the increase in HRQoL research, studies of HRQoL in children have received little attention compared to adults [9]. Furthermore, a large part of the research involving children has focused on clinical groups [5, 9]. A recent review of QoL research in medicine and health sciences revealed that most studies exploring QoL involve adults with a specific disease. Few studies have focused on children and adolescents, and studies including children are mainly clinical and do not involve the general population [9]. This is worrying because knowledge about how children perceive their health and well-being can help to identify individuals at risk of poor HRQoL and further inform the development and evaluation of interventions that may enhance HRQoL for all [3, 8].

The research designs of the selected reports in this study included descriptive, longitudinal, experimental, and case–control designs. Our review shows that KIDSCREEN is increasingly being used as an outcome in population-based studies, often assessed in relation to certain topics or conditions, or as an intervention outcome.

Of the included reports, 9.5% were RCTs, of which 70.8% used KIDSCREEN as a main outcome. Interestingly, 42% of the included RCTs were conducted in 2020 or 2021, which indicates an increase in experimental population-based KIDSCREEN studies. This demonstrates an increased interest in how children and adolescents’ lives are affected by interventions in relation to HRQoL. Further intervention studies are of great importance to the public health field. To measure change in HRQoL over time, additional studies with a longitudinal design are needed [7]. Longitudinal data allow the monitoring of child health development throughout childhood and adolescence to measure changes that occur during development [5] and monitor the effects of interventions [7]. Monitoring HRQoL in children over time complies with the United Nations Sustainable Development Goal 3 of ensuring the well-being of all children [8]. The main increase in KIDSCREEN studies is observed with cross-sectional studies, while a slight increase is seen in longitudinal studies. Despite this increase in longitudinal studies, a further increase in studies investigating the development of HRQoL in children and adolescents across time and with respect to societal changes, adolescent maturation, and life events is needed [5].

Another important finding in our review was that most studies used the self-report version of the KIDSCREEN instrument. This is significant, as self-reporting is the recommended guideline for HRQoL measurement [7, 17]. There is evidence that children can adequately self-report on their health from the age of 8 [54]. A greater number of self-reports may therefore be expected in the age range included in our review. Proxy reporting, however, may be necessary when the child is too young or ill or does not have the necessary language skills, cognitive abilities, or attention span to finish the questionnaire [7]. Only 18 reports in our review provided data on information given by proxy respondents as a supplement to self-reported HRQoL. Of the 20 studies involving the 6–7 age range, only 40% included proxy assessments. Thus, the studies solely relying on subjective data in this age group may have their results questioned, as the self-report versions of the KIDSCREEN instruments are validated from the age of 8 [17]. Children and parents both provide unique information. While children report the here-and-now situation, parents may also consider the future of their child, along with their own well-being influencing the report [7]. Inclusion of both proxy and self-reporting can give a broader picture of children and adolescents’ HRQoL [7]. In the 8–18 age range, as few as 3% of the studies were proxy reported. This aligns with current recommendations of primarily assessing HRQoL subjectively.

The scoring of the KIDSCREEN questionnaires may be done in various ways, and no cut point has yet been developed. The KIDSCREEN manual suggests three different ways to interpret data and calculate threshold scores: comparing group scores and the reference population, using the strengths of the Rasch model (employing person parameter estimates), and interpreting a responder’s KIDSCREEN score by using T-values and percentiles [17]. We found only one study [20] that developed cut points for KIDSCREEN 10. However, the cut points were not developed for use on an individual level. Hence, further work is required regarding the interpretation of KIDSCREEN scores in order to aid healthcare workers in understanding KIDSCREEN data for individual consultations.

Regarding topics assessed in relation to KIDSCREEN, the results reflect the international trends regarding public health challenges. The studies included in our review showed that mental and psychosocial health, physical activity, socioeconomic status, and obesity are most frequently measured in relation to KIDSCREEN. Mental illness among adolescents is an increasing problem and is one of today’s main public health challenges, which is also emphasized by a recent UNICEF report [55]. Mental illness in childhood and adolescence is an indicator of impaired general and mental health 6–11 years later [56]. With the impact of the COVID-19 pandemic, an even greater risk for mental health problems among children and adolescents exists [57]. Moreover, psychosocial distress has a great impact on adolescents’ lives, health, and future circumstances [12, 58]. This represents an increasing challenge that should continue to be investigated in the future [55].

Social inequalities in health also represent a public health concern that appears to have increased over time [59], and the number of children living in families with financial difficulties is expected to increase in developing countries, especially in light of the COVID-19 pandemic [60]. Children living with low SES experience childhood health problems, which may further lead to inadequate health outcomes in adulthood [56, 61]. Assessing SES in relation to KIDSCREEN is of great relevance, as SES is an indicator of the general, mental, and physical health of children and adolescents [61]. Moreover, several studies focused on physical activity and obesity in relation to KIDSCREEN. There is general agreement that regular physical activity has a positive effect on mental health and HRQoL [62, 63], and the World Health Statistics 2021 highlights obesity and physical inactivity as two of the world’s leading causes of death [64]. Consequently, such topics is important to address in HRQoL research among children and adolescents, which is also reflected in our findings.

Knowing of the challenges and changes children and adolescents experience, and considering previous research, we suggest that future research continues to focus on HRQoL assessment in children and adolescents. The WHO has established specific goals of better health and well-being for all people by 2023 [64], and HRQoL assessment in children and adolescents can be an important part of the progress toward this target. Investing in children and adolescents is more effective than investments later in life [61], and HRQoL measurement contributes strongly to such essential investments. Additionally, such knowledge can be used to inform public policy decisions.

## Strengths and limitations

One strength of this scoping review is that we systematically searched relevant databases: Scopus, Embase and MEDLINE, CINAHL, SocINDEX, Eric, and PsycINFO. Another strength is that the selection process, review, and data extraction were performed independently and blinded, in pairs. The involvement and consensus of the six participating researchers contributed to a systematic and transparent review process. One further strength is our results being presented in accordance with our predefined aim. The limitations of this review also deserve to be mentioned. First, while including a large number of studies, we chose broad mapping categories related to KIDSCREEN, which may have diminished more detailed nuances in the studies. However, we chose this approach to best systemize a large number of reports. Second, searches were limited to the English language only. It is possible that relevant reports may have been published in other languages. Twenty-one reports were excluded due to publication in a foreign language. Third, some reports (n = 33) did not mention the age range of the participants. These study participants were therefore not included in Table 3.

## Conclusion

This study was the first review to investigate the use of KIDSCREEN instruments in the general population of children and adolescents. This study demonstrates that the use of KIDSCREEN instruments is increasing in population-based research. We found that KIDSCREEN questionnaires are widely used in cross-sectional studies assessing common public health challenges. Longitudinal studies and RCTs using KIDSCREEN instruments are also increasing. In the 6–18 age range, KIDSCREEN is being used mainly as a self-report instrument. Additionally, experimental, and longitudinal assessments, possibly including relevant cut points, remain mainly unexplored and are recommended for future research.

## Supplementary Information

**Additional file 1**. Scoping review protocol.

**Additional file 2**. Appendix.

## Abbreviations

BMI: Body mass index
HRQoL: Health related quality of life
Prisma-ScR: Preferred Reporting Items for Systematic Reviews and Scoping Reviews
RCTs: Randomized controlled trials
SES: Socioeconomic status
WHO: World Health Organization
