# Supplementary material for: Use of KIDSCREEN health-related quality of life instruments in the general population of children and adolescents: a scoping review
Source: Health Qual Life Outcomes. 2023 Jan 20;21:6. doi: 10.1186/s12955-023-02088-z (PMC9857919; doi:10.1186/s12955-023-02088-z)
Supplement: Supplementary file 1 — Additional file 1. Scoping review protocol. [file 12955_2023_2088_MOESM1_ESM.pdf]

SCOPING REVIEW PROTOCOL

Title: Use of KIDSCREEN health-related quality of life instruments in the general population of children and adolescents - A scoping review

Eva-Grethe Befus1, Sølvi Helseth[1,2], Eirin Mølland[3], Thomas Westergren[1,4], Liv Fegran[1], Kristin Haraldstad[1]

[1] Faculty of Health- and Sport Sciences, University of Agder, P.O. Box 422, 4604 Kristiansand, Norway

[2] Faculty of Health, OsloMet – Oslo Metropolitan University, St. Olavs plass, P.O. Box 4, 0130 Oslo, Norway

[3] Department of Economics and Finance, School of Business and Law, University of Agder, P.O. Box 422, 4604 Kristiansand, Norway

[4] Faculty of Health Sciences, University of Stavanger, P.O. Box 8600, 4036 Stavanger, Norway

Corresponding author: Eva-Grethe Befus, eva-grethe.befus@uia.no

BACKGROUND

In the Norwegian Government's strategy for good mental health for the period 2017-2022, one of the main recommendations is to gain more knowledge on quality of life (QoL) in different parts of the population (1). Mental health problems is regarded as one of the most serious public health concerns of our time (2). According to the World Health Organization (WHO) 10-20 percent of children and adolescents under 18 have mental problems, and the problem is increasing (3). Approximately half of these are assumed to have diagnosable mental illnesses, which require treatment (4). Ensuring good health and improving QoL for all is also one of The United Nation's (UN) sustainability goals (5). Increased knowledge about health related quality of life (HRQoL) will contribute with new content in physical and mental health and encourage development and interventions in public health (6). This review is a part of the *Starting Right* study (https://www.godtbegynt.no) whose goal is to solve health and societal challenges, specified as improving public health, enhancing quality of life, and reducing social inequalities in health. One of the validated instruments used in *Starting right* is the KIDSCREEN 27 questionnaire, which is one of the most used questionnaires for measuring HRQoL in Europe. The KIDSCREEN instrument exists in three different versions, both for children and parents consisting of 52, 27, and 10 items. KIDSCREEN 27 measures physical well-being and psychological well-being in relations to parents, friends, social support and school (7).

HRQoL is a subjective term and multidimensional construct including physiological, psychological, and functional aspects of general well-being (8). The increased focus on children and adolescents' subjective experience represents an evident shift in the last decade, from objective to subjective measures (9)

It is important to assess children and adolescents' HRQoL systematically, to be able to identify children and subgroups that might be at risk of poor HRQoL (9). This is crucial in the trajectory of fulfilling the United Nations Sustainable Development Goal 3 of ensuring good health and improving HRQoL for all (5, 10). Few population-based studies on children and adolescents' HRQoL have been carried out (11). However, numerous studies on cancer and chronic illnesses have been conducted (9). Furthermore, HRQoL has recently become a major health outcome in the public health area (12, 13).

To the best of our knowledge, the application of KIDSCREEN instruments in studies of children and adolescents in the general population have not previously been systematically reviewed. The general population refers to all individuals aged 6–18 years old recruited from a population with no specific disease or clinical condition. However, the KIDSCREEN instrument is increasingly being used in public health- and large-scaled population-based studies. Hence, the need for a review of how the instrument has been used in this context is highlighted.

STUDY AIM

The aim of the present scoping review is to provide an overview of and map studies using KIDSCREEN in the general population of children aged 6–18 years, as well as to describe the country of origin, the study design, whether HRQoL is a main focus, the version of KIDSCREEN instrument(s) being used, the age group, if a cut point for KIDSCREEN is discussed, and the study context.

RESEARCH QUESTIONS

- In which Country is the study performed?
- Which study design is used in the study?
- Is HRQoL a main focus in the study?
- Which version of the KIDSCREEN instruments has been used?
- Which age group is included?
- Does the study describe a cutpoint for HRQoL?
- Which study context was the instrument used?

SEARCH STRATEGY

To prepare the searching process, an identification of the main concept of the study aim was guided by the elements of the PICO structure (14, 15), see table 1.

Table 1

**Population**	Children and adolescents age 6-18 years old
**Interest**	Studies using KIDSCREEN instruments
**Comparison**	----
**Outcome**	In which country is the study performed? Which study design is used in the study? Is HRQoL a main focus in the study? Which version of the KIDSCREEN instruments has been used? Which age group is included? Does the study describe a cutpoint for HRQoL? Which study context was the instrument used?
**Context**	General population

To identify studies using KIDSCREEN questionnaires, a systematic literature search was carried out in the following databases: CINAHL, SocINDEX, MEDLINE, Embase, APA PsychINFO, Eric and Scopus. The databases are chosen to cover a broad sample of the literature. The search strategy will include “kidscreen” as the main keyword, to identify relevant studies. The search will be performed in collaboration with an experienced librarian. The Preferred Reporting Items for Scoping Review checklist will we used to form the base of the upcoming review (16).

INCLUSION AND EXCLUSION CRITERIONS

Eligibility criteria will be conducted to ensure consistency, validity, and reliability.

**INCLUSION**	**EXCLUSION**
Studies using KIDSCREEN instruments in the general population	Studies performed in a clinical setting
If studies do not include “KIDSCREEN” in title or abstract, but “quality of life,” QoL, HRQoL, or “well-being” is present, the full text must be searched to find if KIDSCREEN instruments have been used.	Studies in a foreign language
Children aged 6-18 years old	Conference abstracts
Primary studies/original research publications	Editorials
English language	Opinion articles
	Scientific statement
	Guideline
	Protocol
	Review article

SCREENING PROCESS

Six researchers will take part in the screening process. The screening process will be done in several shifts: 1) Title and abstract will be assessed for inclusion, independently, in pairs. 2) Records in line with our inclusion criteria will be read in full text. This will also be done in pairs. The first author will screen all records, to ensure consistency. Any cases of disagreements during the screening process will de resolved through consensus discussion with all the authors. Rayyan will be used for managing the screening process, and for using the opportunity to "blind on" and ensure consistency between the reviewers. The screening process will be visualised in the PRISMA flow diagram (Figure 1). The aim is to map a topic, not rank it, therefore quality assessment of each study will not be done (17)

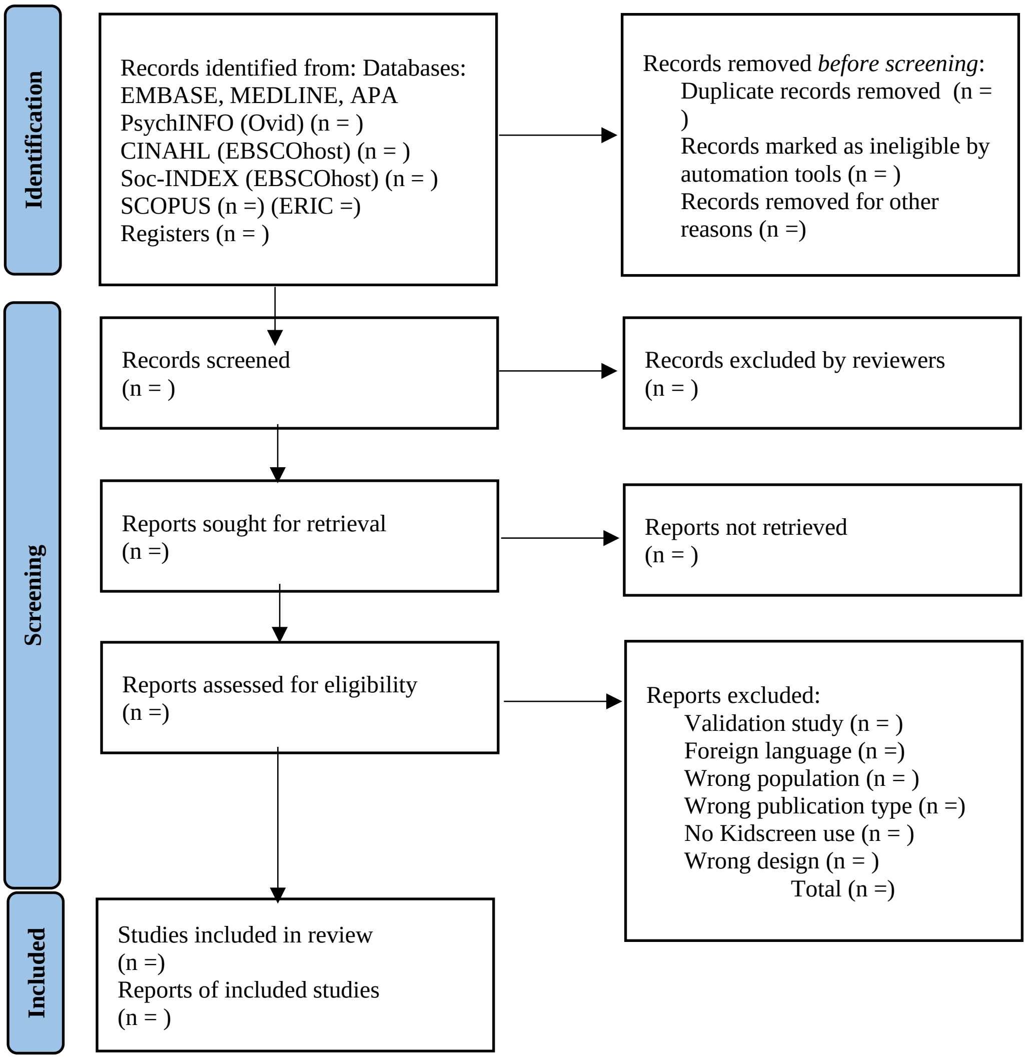

Fig. 1 Flow chart of inclusion. Source: Page MJ, McKenzie JE, Boutron I, Hoffmann TC, Mulrow CD, et. al. The PRISMA 2020 statement: an updated guideline for reporting systematic reviews. BMJ 2021;372:n71. doi: 10.1136/bmj.n71

DATA COLLECTION

The research team will develop the data charting spreadsheet in Excel, and we will test the suitability of the sheet by independently extracting data from 10 articles. After the initial data extraction all authors will discuss the suitability of the spreadsheet and modify it, if necessary. Further, data will be

extracted from individual studies independently, in pairs. The first author will extract data from all included reports.

RESULTS

The results will be presented in text, tables, and figures in line with the aim of the scoping review.

ETHICS AND DISSEMINATION

The upcoming scoping review will follow All European Academies' (ALLEA) four fundamental principles for research integrity: reliability, honesty, respect and accountability (17). Reliability will be ensured using a clearly declared method. The research process will be truthfully described to follow the principle of honesty. The method used in the article will get proper credit, and the investigators will take full responsibility for the studies. The upcoming review will be submitted to a peer reviewed journal.

REFERENCES

1. Regjeringen. Regjeringens strategi for god psykisk helse (2017-2022). Mestre hele livet.; 2017-2022.
2. Bor W, Dean AJ, Najman J, Hayatbakhsh R. Are child and adolescent mental health problems increasing in the 21st century? A systematic review. Aust N Z J Psychiatry. 2014;48(7):606-16.
3. Subjective Economic Status in Adolescence: Determinants

and Associations with Mental Health in the Norwegian Youth@

Hordaland Study [Internet]. Journal of Family and Economic Issues, 1-4. 2017.
4. Kjeldsen A, Nilsen W, Gustavson K, Skipstein A, Melkevik O, Karevold EB. Predicting Well-Being and Internalizing Symptoms in Late Adolescence From Trajectories of Externalizing Behavior Starting in Infancy. J Res Adolesc. 2016

26(4):991-1008.
5. UNICEF. Building the future: Children and the Sustainable Development Goals in Rich Countries 2017 [Available from: https://www.unicef-irc.org/publications/890-building-the-future-children-and-the-sustainable-development-goals-in-rich-countries.html.
6. Bang Nes R, Hansen T, Barstad A. Livskvalitet - Anbefalinger for et bedre målestystem. Oslo: Helsedirektoratet; 2018.
7. Haraldstad K, Richter J. Måleegenskaper ved den norske versjonen av KIDSCREEN. PsykTestBarn. 2014.
8. Otto C, Haller A-C, Klasen F, Hölling H, Bullinger M, Ravens-Sieberer U. Risk and protective factors of health-related quality of life in children and adolescents: Results of the longitudinal BELLA study. 2017.
9. Helseth S, Haraldstad K. Child Well-Being. In: Maggino F, editor. Encyclopedia of Quality of Life and Well-Being Research. Cham: Springer International Publishing; 2020. p. 1-5.
10. Haverman L, Limperg PF, Young NL, Grootenhuis MA, Klaassen RJ. Paediatric health-related quality of life: what is it and why should we measure it? Archives of Disease in Childhood 2017(102):458-.
11. Haraldstad K, Wahl A, Andenaes R, Andersen JR, Andersen MH, Beisland E, et al. A systematic review of quality of life research in medicine and health sciences. Qual Life Res. 2019;28(10):2641-50.
12. Ravens-Sieberer U, Erhart M, Wille N, Wetzel R, Nickel J, Bullinger M. Generic health-related quality-of-life assessment in children and adolescents. Pharmacoeconomics. 2006;24(12):1199-220.
13. Ravens-Sieberer U, Herdman M, Devine J, Otto C, Bullinger M, Rose M, et al. The European KIDSCREEN approach to measure quality of life and well-being in children: development, current application, and future advances. Qual Life Res. 2014;23(3):791-803.
14. Universitetsbiblioteket. Systematiske litteraturstudier-systematiske litteratursøk (HELIDIR).
15. Booth A, Sutton A, Papaioannou D. Systematic approaches to a successful literature review. 2nd ed. ed. Los Angeles, Calif: Sage; 2016.
16. Moher D, Liberati A, Tetzlaff J, Altman DG. Preferred reporting items for systematic reviews and meta-analyses: the PRISMA statement. Int J Surg. 2010;8(5):336-41.
17. Sandberg K, Olsson C, Gjevjon ER, Borglin G. Nursing care and models of care in relation to older people in long-term care contexts: a scoping review protocol. BMJ Open. 2022;12(11):e064610.
