# Supplementary material for: Use of KIDSCREEN health-related quality of life instruments in the general population of children and adolescents: a scoping review
Source: Health Qual Life Outcomes. 2023 Jan 20;21:6. doi: 10.1186/s12955-023-02088-z (PMC9857919; doi:10.1186/s12955-023-02088-z)
Supplement: Supplementary file 2 — Additional file 2. Appendix. [file 12955_2023_2088_MOESM2_ESM.docx]

**APPENDIX**

**EMBASE, MEDLINE, APA PsycINFO (Ovid) SEARCH 211020**

Databases: Embase <1974 to 2020 Week 42>, Ovid MEDLINE(R) ALL <1946 to October 20, 2020>, APA PsycInfo <1806 to October 2020 Week 2>

Search Strategy

1 KIDSCREEN*.mp. (1,657)

2 ((kid or kids*) adj2 screen*).mp. (137)

3 1 or 2 (1785)

4 limit 3 to yr = “2000 -Current” (1,779)

5 remove duplicates from 4 (1,104)

**NOTES**

Proximity – adjacency

- The ADJ2 operator finds terms in any order and with one word (or none) between them.

Field codes Ovid

- .mp (multiple purposes)

**MEDLINE**

**MP**

| **Default Fields for Unqualified Searches (MP):**Searching for a term without specifying a field in Advanced search, or specifying.mp., defaults to the following ‘multi-purpose’ (.mp.) fields for this database: ti,ab,ot,nm,hw,fx,kf,ox,px,rx,ui,sy. | | | |
| --- | --- | --- | --- |
|  | [Abstract](http://ospguides.ovid.com/OSPguides/medline.htm?S=ALGMFPAAPLACAHCPKPAKLHFOFKHMAA00#AB) (AB) | [Organism Supplementary Concept Word](http://ospguides.ovid.com/OSPguides/medline.htm?S=ALGMFPAAPLACAHCPKPAKLHFOFKHMAA00#OX) (OX) | [Subject Heading Word](http://ospguides.ovid.com/OSPguides/medline.htm?S=ALGMFPAAPLACAHCPKPAKLHFOFKHMAA00#HW) (HW) |
|  | [Floating Sub-Heading Word](http://ospguides.ovid.com/OSPguides/medline.htm?S=ALGMFPAAPLACAHCPKPAKLHFOFKHMAA00#FX) (FX) | [Original Title](http://ospguides.ovid.com/OSPguides/medline.htm?S=ALGMFPAAPLACAHCPKPAKLHFOFKHMAA00#OT) (OT) | [Synonyms](http://ospguides.ovid.com/OSPguides/medline.htm?S=ALGMFPAAPLACAHCPKPAKLHFOFKHMAA00#SY) (SY) |
|  | [Keyword Heading Word](http://ospguides.ovid.com/OSPguides/medline.htm?S=ALGMFPAAPLACAHCPKPAKLHFOFKHMAA00#KF) (KF) | [Protocol Supplementary Concept Word](http://ospguides.ovid.com/OSPguides/medline.htm?S=ALGMFPAAPLACAHCPKPAKLHFOFKHMAA00#PX) (PX) | [Title](http://ospguides.ovid.com/OSPguides/medline.htm?S=ALGMFPAAPLACAHCPKPAKLHFOFKHMAA00#TI) (TI) |
|  | [Name of Substance Word](http://ospguides.ovid.com/OSPguides/medline.htm?S=ALGMFPAAPLACAHCPKPAKLHFOFKHMAA00#NM) (NM) | [Rare Disease Supplementary Concept Word](http://ospguides.ovid.com/OSPguides/medline.htm?S=ALGMFPAAPLACAHCPKPAKLHFOFKHMAA00#RX) (RX) | [Unique Identifier](http://ospguides.ovid.com/OSPguides/medline.htm?S=ALGMFPAAPLACAHCPKPAKLHFOFKHMAA00#UI) (UI) |
|  | | | |

**PsycINFO**

| **Default Fields for Unqualified Searches (MP):**Searching for a term without specifying a field in Advanced search, or specifying.mp., defaults to the following ‘multi-purpose’ (.mp.) fields for this database: ti,ab,hw,tc,id,ot,tm,mh. | | | | |
| --- | --- | --- | --- | --- |
|  | [Abstract](http://ospguides.ovid.com/OSPguides/psycdb.htm?S=ALGMFPAAPLACAHCPKPAKLHFOFKHMAA00#AB) (AB) | [Key Concepts](http://ospguides.ovid.com/OSPguides/psycdb.htm?S=ALGMFPAAPLACAHCPKPAKLHFOFKHMAA00#ID) (ID) | [Original Title](http://ospguides.ovid.com/OSPguides/psycdb.htm?S=ALGMFPAAPLACAHCPKPAKLHFOFKHMAA00#OT) (OT) | [Test & Measures](http://ospguides.ovid.com/OSPguides/psycdb.htm?S=ALGMFPAAPLACAHCPKPAKLHFOFKHMAA00#TM) (TM) |
|  | [Heading Word](http://ospguides.ovid.com/OSPguides/psycdb.htm?S=ALGMFPAAPLACAHCPKPAKLHFOFKHMAA00#HW) (HW) | [MeSH](http://ospguides.ovid.com/OSPguides/psycdb.htm?S=ALGMFPAAPLACAHCPKPAKLHFOFKHMAA00#MH) (MH) | [Table of Contents](http://ospguides.ovid.com/OSPguides/psycdb.htm?S=ALGMFPAAPLACAHCPKPAKLHFOFKHMAA00#TC) (TC) | [Title](http://ospguides.ovid.com/OSPguides/psycdb.htm?S=ALGMFPAAPLACAHCPKPAKLHFOFKHMAA00#TI) (TI) |

| **ID** | **Key Concepts [Word Indexed] bipolar.id. psychosis.id.** |
| --- | --- |
|  | The Key Concepts (ID) field concisely summarizes a document’s subject content. Indexers use the Key Concepts to supplement [Subject Headings](http://ospguides.ovid.com/OSPguides/psycdb.htm?S=ALGMFPAAPLACAHCPKPAKLHFOFKHMAA00#SH) (SH).  For experimental literature, Key Concepts typically contain the independent variable, the dependent variable, and the subject population.  For nonexperimental literature, Key Concepts consist of major concepts, timelines, populations, implications, or genres, whatever information the indexer thinks will supplement other indexing information.  Individual words from the Key Concepts may be searched in this field. |

| **TM** | **Test & Measures [Word Indexed] interview.tm.** |
| --- | --- |
|  | The Test & Measures (TM) field identifies the names of tests and measures mentioned in the source document. |

**EMBASE**

| **Default Fields for Unqualified Searches (MP):**Searching for a term without specifying a field in Advanced search, or specifying.mp., defaults to the following “multi-purpose” (.mp.) fields for this database: ti,ab,hw,tn,ot,dm,mf,dv,kw,fx,dq. | | | |
| --- | --- | --- | --- |
|  | [Abstract](http://ospguides.ovid.com/OSPguides/embase.htm?S=ALGMFPAAPLACAHCPKPAKLHFOFKHMAA00#ab) (AB) | [Drug Manufacturer](http://ospguides.ovid.com/OSPguides/embase.htm?S=ALGMFPAAPLACAHCPKPAKLHFOFKHMAA00#mf)(MF) | [Keyword](http://ospguides.ovid.com/OSPguides/embase.htm?S=ALGMFPAAPLACAHCPKPAKLHFOFKHMAA00#kw) (KW) |
|  | [Candidate Term Word](http://ospguides.ovid.com/OSPguides/embase.htm?S=ALGMFPAAPLACAHCPKPAKLHFOFKHMAA00#dq) (DQ) | [Drug Trade Name](http://ospguides.ovid.com/OSPguides/embase.htm?S=ALGMFPAAPLACAHCPKPAKLHFOFKHMAA00#tn) (TN) | [Original Title](http://ospguides.ovid.com/OSPguides/embase.htm?S=ALGMFPAAPLACAHCPKPAKLHFOFKHMAA00#ot) (OT) |
|  | [Device Manufacturer](http://ospguides.ovid.com/OSPguides/embase.htm?S=ALGMFPAAPLACAHCPKPAKLHFOFKHMAA00#dm) (DM) | [Floating Subheading Word](http://ospguides.ovid.com/OSPguides/embase.htm?S=ALGMFPAAPLACAHCPKPAKLHFOFKHMAA00#fx) (FX) | [Title](http://ospguides.ovid.com/OSPguides/embase.htm?S=ALGMFPAAPLACAHCPKPAKLHFOFKHMAA00#ti) (TI) |
|  | [Device Trade Name](http://ospguides.ovid.com/OSPguides/embase.htm?S=ALGMFPAAPLACAHCPKPAKLHFOFKHMAA00#dv) (DV) | [Heading Word](http://ospguides.ovid.com/OSPguides/embase.htm?S=ALGMFPAAPLACAHCPKPAKLHFOFKHMAA00#hw) (HW) |  |

| **KW** | **Keyword [Word Indexed] biomarkers.kw.** |
| --- | --- |
|  | This Keyword field (KW) contains keywords defined by the author of the article. |

**CINAHL (EBSCOhost) 211020**

**NOTE:** The default fields for unqualified searches consist of the following: title, abstract and subject headings.

| **#** | **Query** | **Limiters/Expanders** | **Last Run Via** | **Results** |
| --- | --- | --- | --- | --- |
| S4 | S1 OR S2 | Limiters – Peer Reviewed; Published Date: 20000101– Search modes – Boolean/Phrase | Interface – EBSCOhost Research Databases Search Screen – Advanced Search Database – CINAHL | 255 |
| S3 | S1 OR S2 | Search modes – Boolean/Phrase | Interface – EBSCOhost Research Databases Search Screen – Advanced Search Database – CINAHL | 268 |
| S2 | (kid OR kids) N1 screen* | Search modes – Boolean/Phrase | Interface – EBSCOhost Research Databases Search Screen – Advanced Search Database – CINAHL | 30 |
| S1 | KIDSCREEN* | Search modes – Boolean/Phrase | Interface – EBSCOhost Research Databases Search Screen – Advanced Search Database – CINAHL | 238 |

**SCOPUS**

Advanced search

TITLE-ABS-KEY(KIDSCREEN*) OR TITLE-ABS-KEY((kid OR kids) w/2 screen*)

646 hits

**Soc-INDEX (EBSCOhost)**

The default fields for unqualified searches consist of the following: all authors, all subjects, all keywords, all title info (including source title), and all abstracts.

| # | Query | Limiters/Expanders | Last Run Via | Results |
| --- | --- | --- | --- | --- |
| S4 | S1 OR S2 | Limiters – Peer Reviewed; Published Date: 20000101- Search modes - Boolean/Phrase | Interface – EBSCOhost Research Databases Search Screen – Advanced Search Database – SocINDEX | 31 |
| S3 | S1 OR S2 | Search modes – Boolean/Phrase | Interface – EBSCOhost Research Databases Search Screen – Advanced Search Database – SocINDEX | 31 |
| S2 | (kid OR kids) N1 screen* | Search modes – Boolean/Phrase | Interface – EBSCOhost Research Databases Search Screen – Advanced Search Database – SocINDEX | 2 |
| S1 | KIDSCREEN* | Search modes – Boolean/Phrase | Interface – EBSCOhost Research Databases Search Screen – Advanced Search Database – SocINDEX | 29 |

# Results

- Total hits: 2,414
- Duplicates – removed through EndNote: 1,048
- Unique hits, screening title/abstracts: 1,366
